# AG1^®^ Induces a Favorable Impact on Gut Microbial Structure and Functionality in the Simulator of Human Intestinal Microbial Ecosystem^®^ Model

**DOI:** 10.3390/cimb46010036

**Published:** 2024-01-05

**Authors:** Trevor O. Kirby, Philip A. Sapp, Jeremy R. Townsend, Marlies Govaert, Cindy Duysburgh, Massimo Marzorati, Tess M. Marshall, Ralph Esposito

**Affiliations:** 1 Research, Nutrition, and Innovation, Athletic Greens International, Carson City, NV 89701, USA; philip.sapp@athleticgreens.com (P.A.S.); jeremy.townsend@athleticgreens.com (J.R.T.); tess.marshall@athleticgreens.com (T.M.M.); ralph.esposito@athleticgreens.com (R.E.); 2 Health & Human Performance, Concordia University Chicago, River Forest, IL 60305, USA; 3 ProDigest BVBA, B-9052 Ghent, Belgium; marlies.govaert@prodigest.eu (M.G.); cindy.duysburgh@prodigest.eu (C.D.); massimo.marzorati@prodigest.eu (M.M.); 4 Center of Microbial Ecology and Technology (CMET), Ghent University, B-9000 Ghent, Belgium; 5 Department of Nutrition, Food Studies, and Public Health, New York University-Steinhardt, New York, NY 10003, USA

**Keywords:** foundational nutrition, metagenomics, microbiome, prebiotics, *Faecalibacterium prausnitzii*

## Abstract

Modulation of the human gut microbiome has become an area of interest in the nutraceutical space. We explored the effect of the novel foundational nutrition supplement AG1^®^ on the composition of human microbiota in an in vitro experimental design. Employing the Simulator of Human Intestinal Microbial Ecosystem (SHIME^®^) model, AG1^®^ underwent digestion, absorption, and subsequent colonic microenvironment simulation under physiologically relevant conditions in healthy human fecal inocula. Following 48 h of colonic simulation, the gut microbiota were described using shallow shotgun, whole genome sequencing. Metagenomic data were used to describe changes in community structure (alpha diversity, beta diversity, and changes in specific taxa) and community function (functional heterogeneity and changes in specific bacterial metabolic pathways). Results showed no significant change in alpha diversity, but a significant effect of treatment and donor and an interaction between the treatment and donor effect on structural heterogeneity likely stemming from the differential enrichment of eight bacterial taxa. Similar findings were observed for community functional heterogeneity likely stemming from the enrichment of 20 metabolic pathways characterized in the gene ontology term database. It is logical to conclude that an acute dose of AG1 has significant effects on gut microbial composition that may translate into favorable effects in humans.

## 1. Introduction

Prebiotics were first defined in 1995 by Gibson and Roberfroid as being nondigestible food ingredients that beneficially impact the host through selective stimulation of beneficial colonic bacteria [1]. Interestingly, this first definition restricted the concept of prebiotics to a select few carbohydrate compounds, namely β-fructans and galacto-oligosaccharides [2]. The concept of what qualifies as a prebiotic has evolved since 1995 and experimental evidence suggests that other phytochemicals beyond just fibers can act as prebiotics [3,4,5]. Phytochemicals behave much like prebiotics in the fact that they are non-digestible food components and exert health benefits through the modulation of the gut microbiota [6,7,8]. Interestingly, phytochemicals can act like traditional prebiotics when they contain glycosidic residues as the glycosides can be cleaved from the parent molecule and undergo fermentation [4,8]. However, many other phytochemicals without clear glycosidic residues can be metabolized by gut microbiota to yield metabolites, resulting in favorable health benefits to the host [9]. Regardless of this, the health benefits largely come from favorable metabolite formation as well as significant modifications to the intestinal microbial ecology [7].

Previously, we described the prebiotic potential of the novel foundational nutrition product known as AG1^®^ (AG1). We showed that AG1 had the potential to demonstrate physical indications of fermentation and increase the production of short-chain fatty acids (SCFAs) [10]. From these data, it is clear that the chemical composition of AG1 is fermentable and could likely exert a prebiotic effect on human gut microbiota. AG1 contains various phytonutrients (e.g., fibers, polyphenols, etc.), which could act as prebiotics from adaptogens, like ashwagandha (*Withania somnifera*) and functional mushrooms [e.g., shiitake (*Lentinula edodes*) and reishi (*Ganoderma lucidum*)], two probiotics (*Lactobacillus acidophilus* UALa-01 and *Bifidobacterium bifidum* UABb-10), and various vitamins and minerals [11]. Prebiotics, probiotics, and micronutrients have all been shown to exert beneficial effects on the gut microbiome [12,13,14].

Considering previous experimental data on AG1 and the growing evidence that various phytochemicals can act as prebiotics [3,4,5], the primary objective of the current experiment was to detail the specific effects of AG1 on gut microbial community composition by observing changes in community structure and function. Using the in vitro experimental design of the Simulator of Human Intestinal Microbial Ecosystem (SHIME^®^) model, we sought to examine how AG1 shifts gut microbial community dynamics through shallow whole genome sequencing (WGS) via the shotgun sequencing methodology approach. This approach allows for observations of in-depth changes in community structure and function resulting from an acute treatment with a novel nutraceutical, AG1 [15]. We hypothesized that AG1-treated stool inocula would exhibit favorable shifts consistent with a synbiotic treatment.

## 2. Materials and Methods

### 2.1. Experimental Product and Model

AG1^®^ (AG1; Athletic Greens International, Carson City, NV, USA) is a novel foundational nutrition supplement containing a mixture of vitamins, minerals, prebiotics, probiotics, and phytonutrients. A recommended dose of AG1 designed for human consumption is 12 g per serving. For the current experiment, a dose of 6 g/reactor was chosen to mitigate physical complications that would impact the mechanical and biological factors of the SHIME^®^ model. The placebo group only received the blank control medium used to deliver AG1. The ingredients in AG1 are available online [11] and have undergone evaluation and verification via NSF testing (Ann Arbor, MI, USA) to ensure the product meets strict quality, purity, safety, and label accuracy standards [16].

Briefly, we employed the SHIME^®^ model, which is jointly registered by ProDigest and Ghent University in Belgium [17]. This model was chosen as it emulates the chemical and physiological conditions of the human gastrointestinal tract to simulate realistic conditions anticipated in humans. Employing this model allows for consistent digestive conditions and controls for experimental variables that could differ significantly when carried out in humans, confounding the potential results. AG1 was exposed to a gastric phase in which the test product was subjected to normal stomach physiological conditions. Following the gastric phase, physiological conditions were shifted toward conditions of the duodenum briefly and then transferred to a dialysis membrane to emulate absorption of the digested fraction. The non-digested fraction was subsequently transferred to a mixture of a colonic medium and human fecal inocula. Human fecal inocula was derived from three independent and seemingly healthy human donors. Health status was determined using the study site and the testimonies provided by donors. Colonic simulations were performed under physiological conditions of the proximal colon for 48 h. For more detailed information on the methodology, please refer to our previous publication [10]. The study was conducted in accordance with the Declaration of Helsinki and approved by the Ethics Committee of the University Hospital Ghent (reference number: ONZ-2022-0267).

### 2.2. DNA Extraction, Library Preparation, and Sequencing

Genomic DNA was extracted from samples taken at the start (0 h) as well as after 48 h of colonic simulation using the CTAB-Phenol-Chloroform methodology [18]. After final precipitation, the DNA was resuspended in PCR water and stored at −20 °C for a further analysis.

Genomic DNA samples were profiled with shotgun metagenomic sequencing. Sequencing libraries were prepared using the Nextera XT DNA Library Preparation Kit (Illumina, San Diego, CA, USA) and IDT Unique Dual Indexes with a total of 1 ng DNA input following the manufacturer’s protocol. Genomic DNA was fragmented using a proportional amount of the Illumina Nextera XT fragmentation enzyme. Unique dual indexes were added to each sample followed by 12 cycles of PCR to construct libraries. DNA libraries were purified using AMpure magnetic Beads (Beckman Coulter, Brea, CA, USA) and eluted in an EB buffer (QIAGEN, Hilden, Germany). DNA libraries were quantified using a Qubit 4 fluorometer and Qubit dsDNA HS Assay Kit. The final pool was quantified using qPCR using the Qubit 4 fluorometer and Qubit™ dsDNA HS Assay Kit (Thermo Fisher Scientific, Waltham, MA, USA). Libraries were then sequenced on the Illumina NovaSeq platform, 2 × 150 bp.

### 2.3. Bioinformatics

A bioinformatic analysis was performed using CosmosID-HUB software version 1.0.0. Initial QC, adapter trimming, and preprocessing of metagenomic sequencing reads were carried out using BBduk [19]. The system utilizes a high-performance data mining k-mer algorithm that rapidly disambiguates millions of short sequence reads into the discrete genomes engendering the particular sequences. The pipeline has two separable comparators: the first consists of a precomputation phase for reference databases and the second is a per-sample computation. The input to the precomputation phase is databases of reference genomes, virulence markers, and antimicrobial resistance markers that are continuously curated by CosmosID scientists. The output of the precomputational phase is a phylogeny tree of microbes, together with sets of variable-length k-mer fingerprints (biomarkers) uniquely associated with distinct branches and leaves of the tree. The second per-sample computational phase searches the hundreds of millions of short sequence reads, or alternatively contigs from draft de novo assemblies, against the fingerprint sets. This query enables the sensitive yet highly precise detection and taxonomic classification of microbial NGS reads. The resulting statistics are analyzed to return the fine-grain taxonomic and relative abundance estimates for the microbial NGS datasets. To exclude false positive identifications, the results are filtered using a filtering threshold derived based on internal statistical scores that are determined by analyzing a large number of diverse metagenomes. Relative abundances were calculated by dividing the specific count of a taxon by the total number of reads per sample. No transformations were applied. An unsupervised reduction technique was applied to only explore bacterial taxa with filtered results, which only contained calls that met the threshold for high confidence on the organism called within the sample.

The quality-controlled reads were then subjected to a translated search against a comprehensive and non-redundant protein sequence database, UniRef 90. The UniRef 90 database, provided by UniProt [20], represents a clustering of all non-redundant protein sequences in UniProt, such that each sequence in a cluster aligns with 90% identity and 80% coverage of the longest sequence in the cluster. The mapping of metagenomic reads to gene sequences was weighted by mapping quality, coverage, and gene sequence length to estimate community-wide weighted gene family abundances as described by Franzosa et al. [21]. Gene families are then annotated to MetaCyc reactions (Metabolic Enzymes) to reconstruct and quantify MetaCyc metabolic pathways [22] in the community as described by Franzosa et al. [21]. The UniRef 90 gene families were also regrouped to Enzyme Commission Enzymes, Pfam protein domains, CAZy enzymes, and GO Terms to obtain an exhaustive overview of gene functions in the community. Lastly, to facilitate comparisons across multiple samples with different sequencing depths, the abundance values are normalized using total sum scaling (TSS) normalization to produce copies per million (CPM) units.

### 2.4. Statistics

Statistical calculations were performed using in-house R-based applications by ProDigest. All analyses were run in R 4.2.2 (https://www.r-project.org/) [23]. Species relative abundance data were used to calculate the Shannon Diversity, Chao1 Diversity, and Simpson Diversity indices. Alpha diversity metrics were calculated using the phyloseq (https://github.com/joey711/phyloseq) and vegan (https://cran.r-project.org/web/packages/vegan/index.html) packages in R. Differences between the AG1-treated microbiota and the blank-control-treated microbiota were assessed after 48 h of colonic simulation via the paired *t*-test. Compositional heterogeneity in the community structure data was determined using the Bray–Curtis Dissimilarity Index and statistically evaluated using the *adonis2* function in the vegan R package. Adonis is a permutational multivariate analysis of variance (PERMANOVA) using Bray–Curtis and Jaccard distance measures to assess microbial community compositional differences when different categorical groups are provided. A total of 1000 permutations were performed, and the adjusted *p*-values are reported. To visualize the compositional heterogeneity, partial least squares-discriminate analyses (PLS-DA) were used using the *plsda* and *plotIndiv* functions in the mixOmics R package (http://mixomics.org/). The linear discriminant effect size (LEfSe) method was used to identify the key bacterial groups that likely accounted for heterogeneity in the community due to being statistically enriched in one of the treatment groups relative to the other. Differences between the AG1-treated communities and the blank-treated communities after 48 h of colonic simulation were analyzed via a linear discriminant analysis (LDA).

Similar methodology and subsequent statistical analyses were also performed to assess compositional heterogeneity in the community functionality data. This methodology was applied to both the gene ontology (GO) terms as well as the MetaCyc pathway database.

## 3. Results

### 3.1. Community Structure

#### 3.1.1. Alpha Diversity

**Figure 1:**
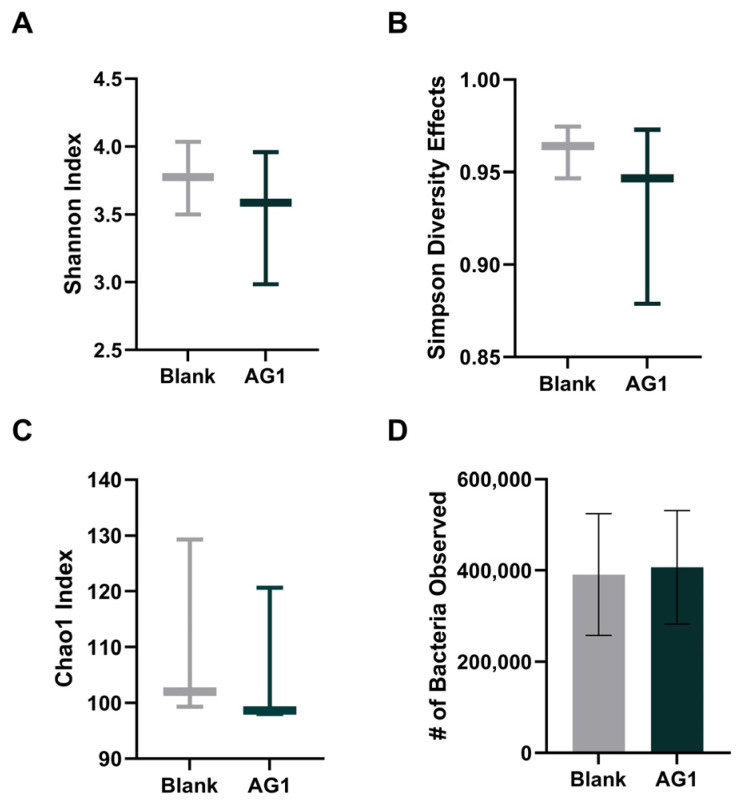
Alpha diversity measures and total observed bacterial abundance after 48 h of colonic simulation. (**A**) Effect of AG1 on Shannon Diversity Index; (**B**) effect of AG1 on Simpson’s Diversity Effects; (**C**) effect of AG1 on Chao1 Diversity Index; (**D**) effect of AG1 on total bacterial abundance.

To observe changes in community structure, changes in alpha diversity were examined (Figure 1). To ensure species richness and evenness, rare taxa were all taken into consideration, and the Shannon Diversity Index, the Simpson’s Diversity Effects, and the Chao1 Diversity Index were calculated for both the AG1-treated microbiota as well as the blank-control-treated microbiota following 48 h of colonic simulation. All three measures failed to reach statistical significance (*p* = 0.1876, *p* = 0.2845, *p* = 0.1794, respectively). Further, the total observed bacterial abundance was analyzed, but failed to reach statistical significance (*p* = 0.2608).

#### 3.1.2. Beta Diversity

**Figure 2:**
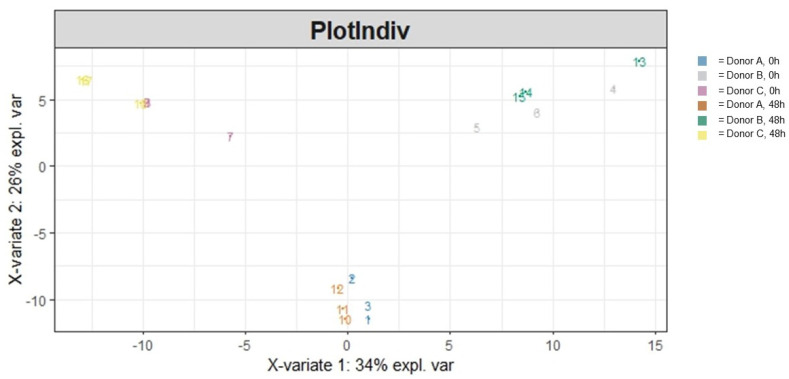
A PLS-DA ordination visualizing the effects of treatment and donor influencing community structural heterogeneity following 48 h of colonic simulation. Numbers 1 through 9 refer to AG1-treated samples and numbers 10 through 18 refer to blank-control-treated samples. Numbers 1 through 3 and 10 through 12 refer to Donor A, numbers 4 through 6 and 13 through 15 refer to Donor B, and numbers 7 through 9 and 16 through 18 refer to Donor C.

Overall heterogeneity in the community structure was visualized (Figure 2) and statistically evaluated. Ordination (PLS-DA) of the data demonstrated a clear donor and treatment effect. Running an adonis (Table 1) to statistically evaluate the variation in the community structure showed that both the treatment and specific donor source significantly impacted overall community heterogeneity (*p* < 0.001 and *p* < 0.001, respectively) and that the interaction between those two variables was significant as well (*p* < 0.001). Moreover, a majority (79.1%) of the variation in community heterogeneity was explained by the donor, followed by the treatment (7.0%), and the interaction between the two variables explained 8.2% of the variation in community heterogeneity.

#### 3.1.3. Specific Taxonomic Differences

**Figure 3:**
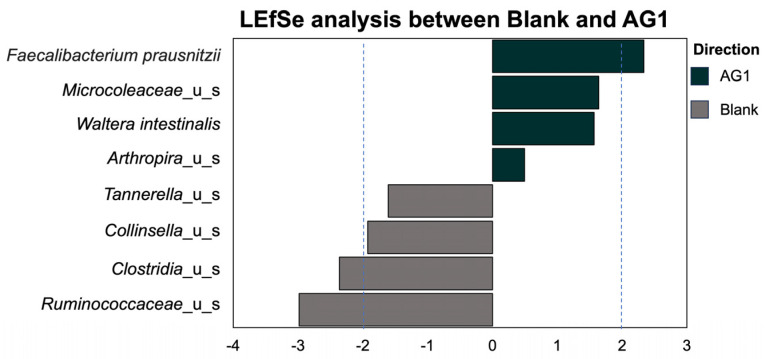
The enrichment of several significant taxa was identified and was dependent on treatment type.

**Figure 4:**
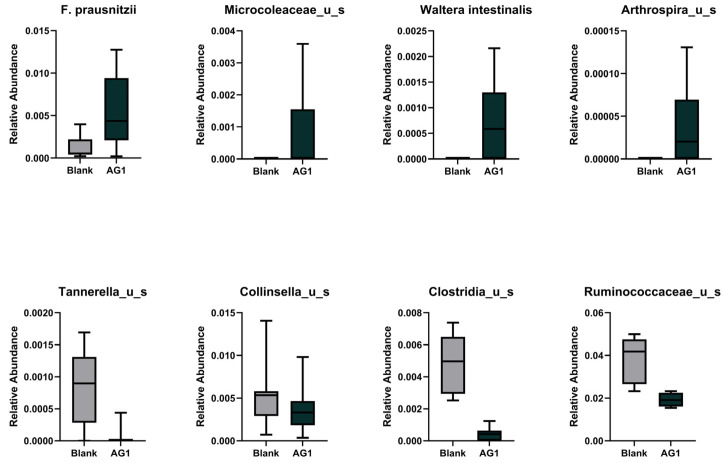
The relative abundances of the taxa captured in the LEfSe analysis for the 48 h time point.

We extended the specific taxonomical investigations to all phylogenetic levels. Using the LEfSe methodology and subsequent LDA (Figure 3), we identified eight statistically significant taxa that differed between the AG1- and blank-control-treated microbiota. Of these, *Faecalibacterium prausnitzii*, *Microcoleaceae*, *Waltera intestinalis*, and *Arthrospira* were enriched in the AG1-treated samples. Conversely, *Tannerella*, *Collinsella*, *Clostridia*, and *Ruminococcaceae* were enriched in the blank-treated samples (Figure 4).

### 3.2. Community Function

#### 3.2.1. Community Functional Heterogeneity

**Figure 5:**
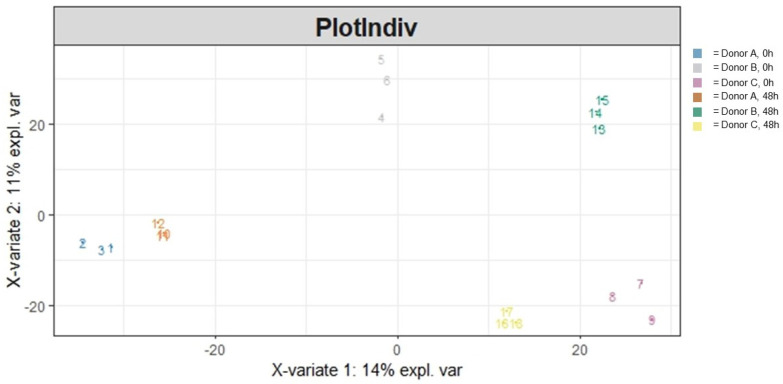
A PLS-DA ordination visualizing the effects of treatment and donor influencing community functional heterogeneity using the GO Term database following 48 h of colonic simulation. Numbers 1 through 9 refer to AG1-treated samples and numbers 10 through 18 refer to blank-control-treated samples. Numbers 1 through 3 and 10 through 12 refer to Donor A, numbers 4 through 6 and 13 through 15 refer to Donor B, and numbers 7 through 9 and 16 through 18 refer to Donor C.

**Figure 6:**
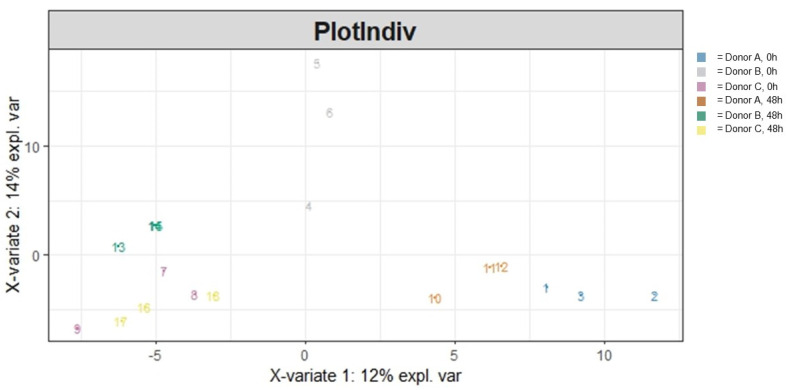
A PLS-DA ordination visualizing the effects of treatment and donor influencing community functional heterogeneity using the MetaCyc database following 48 h of colonic simulation. Numbers 1 through 9 refer to AG1-treated samples and numbers 10 through 18 refer to blank-control-treated samples. Numbers 1 through 3 and 10 through 12 refer to Donor A, numbers 4 through 6 and 13 through 15 refer to Donor B, and numbers 7 through 9 and 16 through 18 refer to Donor C.

To understand the general differences in how the microbial community was functioning due to AG1 treatment after 48 h of colonic simulation, functional heterogeneity was visualized via ordination and subsequently statistically evaluated. This was run for both GO Terms (Figure 5) as well as for data using the MetaCyc database (Figure 6). For the GO Terms (Table 2), there was a significant treatment effect (*p* = 0.034) and donor effect (*p* < 0.001), with a significant interaction between the two variables (*p* = 0.025). A majority (43.6%) of the variation in community functional heterogeneity was explained by the donor, followed by the treatment (7.1%), and the interaction between the two variables explained 12.1% of the variation in community functional heterogeneity. Results for the MetaCyc database were different (Table 3). There was no significant treatment effect (*p* = 0.051). However, there was a clear donor effect (*p* < 0.001) but no interaction between the treatment effect and donor (*p* = 0.496). A majority (24.4%) of the variation in community functional heterogeneity was explained by the donor, followed by the treatment (8.6%), and the interaction between the two variables explained 9.3% of the variation in community functional heterogeneity.

#### 3.2.2. Specific Community Functional Pathways

**Figure 7:**
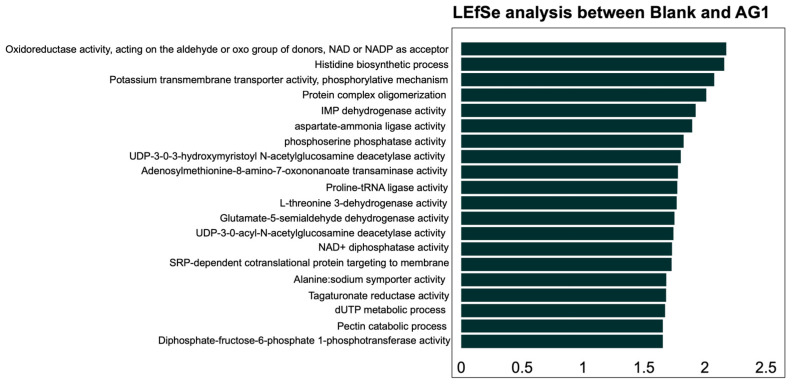
The enrichment of several significant functional pathways was identified using the GO Term database and was dependent on treatment with AG1. All pathways enriched were observed only in the AG1-treated samples.

**Figure 8:**
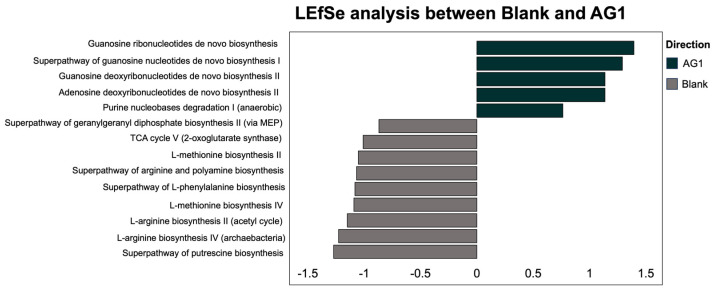
The enrichment of several significant functional pathways was identified using the MetaCyc database and was dependent on treatment type.

Using the LEfSe methodology and subsequent LDA statistical analysis, we identified 20 functional pathways from the GO Terms that were explicitly enriched in the AG1-treated microbiota communities and no pathways that were enriched in the blank-control-media-treated microbiota communities (Figure 7). For the MetaCyc pathway database, a total of five functional pathways were enriched in the AG1-treated samples while nine functional pathways were enriched in blank-control-media-treated samples (Figure 8).

## 4. Discussion

From the totality of the data, it is reasonable to conclude that an acute dose of AG1 has an overall significant impact on the gut microbiome’s community composition. Regarding community structure, we did not observe a significant impact on alpha diversity, but did see a significant treatment effect and donor effect, and an interaction between the treatment and donor effect on structural heterogeneity. Several significant taxa were enriched or diminished by the AG1 treatment and explain the significant heterogeneity observed. Further, similar findings were observed regarding community functional heterogeneity. There was a significant treatment effect and donor effect, and an interaction between the treatment and donor effect on functional heterogeneity that corresponded to significantly enriched functional pathways.

Total abundance, Shannon Diversity, Simpson’s Diversity Effects, and the Chao1 Diversity Index all failed to demonstrate significant changes in community diversity. Despite this, there was a general trend that AG1 supplementation reduced community diversity. While increased alpha diversity is generally a beneficial phenomenon [24], species diversity tends to decrease during succession [25]. This occurrence is likely attributed to the primary succession process observed when the bioreactors were seeded with fecal inocula. In a chronic paradigm, this phenomenon would likely not be observed as diversity tends to increase after primary succession, at least in plant communities [26,27]. Further, treatment with prebiotics might have also impacted alpha diversity. It is implied that prebiotics can reduce alpha diversity by favorably selecting bacteria capable of fermentation, resulting in decreased species richness and evenness, especially in vitro [28,29,30]. These theories are probable as we also saw a generalized increase in the total number of bacteria observed. Prebiotic interventions have been shown to increase relative abundances in gut microbial communities [31].

We then turned to look at beta diversity, which measures variation in taxonomic composition in a community rather than just taxonomic richness or evenness [32]. When investigating the community structure accounting for the dependence each taxon has on one another, we were able to see the significant heterogeneity within the AG1-treated communities relative to the blank-control-treated communities. The heterogeneity in community structure was driven by the enrichment and diminishment of specific taxa caused by AG1 supplementation. Of the enriched taxa, two were likely transient microbiota and two were resident microbiota. The two likely transient taxa, *Microcoleaceae* and *Arthrospira*, were cyanobacteria in nature. Interestingly, the genus *Arthrospira* encompasses Spirulina. AG1 contains various whole-food products including Spirulina; it is theorized that these two taxa were found to be enriched in the microbial communities treated with AG1 simply because these microbes entered the colon microenvironment from the test product. The two resident taxa were *F. prausnitzii* and *W. intestinalis*. *F. prausnitzii* is a well-known microbe with many purported health benefits due to its ability to synthesize butyrate [33,34,35]. Therefore, increased *F. prausnitzii* is promising and has been shown to increase in abundance when prebiotics are taken [36]. *W. intestinalis* is a prevalent gut bacterial species in humans with a distant relation to *Kineothrix alysoides*. Genetically, *W. intestinalis* is suggested to have a diverse metabolic potential within the microbiome [37]. Moreover, *W. intestinalis* harbors genes for the biosynthesis of Ruminococcin A, which has anti-*Clostridium* spp. activity [38].

It is then not surprising that *Clostridia* was not enriched in the AG1-treated samples. However, decreases in *Clostridia* make it hard to ascertain the repercussions in humans as the taxon is incredibly large and diverse [39]. Beyond *Clostridia*, *Tannerella* and *Collinsella* were also shown to decrease in AG1-treated microbial communities. The genus *Tannerella* is known for its oral pathogen *Tannerella forsythia*, and, although beyond the scope of the current experiment, prebiotics have been shown to lower *Tannerella* and improve lipid metabolism in mice [40]. Decreases in *Collinsella* have clear implications for human health as increased *Collinsella* abundances are linked with low fiber intake and correlated to circulating insulin [41], in increased abundances in the gut microbiome of patients with atherosclerosis [42], shown to increase gut permeability and be associated with rheumatoid arthritis [43], and known to be proinflammatory and a risk factor for Alzheimer’s disease [44]. The last taxa shown to be decreased in the AG1-treated samples was *Ruminococcacea*, which was surprising. Generally, *Ruminococcaceae* has been shown to be associated with health benefits [45,46]. To fully understand the biological ramifications of AG1, the gut microbiome needs to be evaluated in humans, in sufficient sample sizes, and in a chronic/long-term dosing paradigm.

Lastly, we sought to look at how changes in community structure translated into changes in the microbiome’s functionality. The effect of AG1 on community functional heterogeneity was significant using the GO Terms and close to significance when using the MetaCyc database. Interestingly, the ordination of community functionality showed that the magnitude of the treatment was highly donor-specific. Regardless, the two databases suggested that AG1 enriched different genes and likely resulted in different metabolic potential within the microbiome. In both databases, the genes appeared to be potentially related to protein biosynthesis, transcription, and translation, and DNA synthesis and replication. The ultimate biological effects of these genetic processes on a host are not able to be determined in an in vitro experimental design and further testing must be performed in a clinical setting to evaluate any effect if there is one at all.

This study had several limitations that warrant interpretation of the data to be taken with caution. The primary limitation is the small sample size of the study given the fact that the human microbiome is incredibly diverse and metabolically complex, leading to large amounts of variability [47,48,49,50]. Further, this is an in vitro model of the human gastrointestinal tract and only a simulation of the colonic environment. Many dynamics that occurred within the community could play out differently in vivo. Finally, the ecological parameters could have biased some of the experimental outcomes. Namely, since fresh stool inocula were used to seed the bioreactors, the community was put under a primary succession event. A chronic dosing paradigm should be used in the future to account for succession (whether it is primary or secondary) and to account for a more longitudinal effect of AG1 on the gut microbiome. Further, pooling of donor stool samples to mitigate individuality and creating a “universal” microbiome could be a solution to mitigate the massive variation between donors but would come with unique benefits and shortcomings with its interpretation. Despite these limitations, the SHIME^®^ model does an impressive job emulating the physiological and biological environment of the digestive tract. Therefore, the current data are invaluable for future, subsequent clinical studies.

## 5. Conclusions

The objective of the study was to observe changes in human gut microbial community composition in an in vitro setting. Data showed that AG1 had no overall effect on alpha diversity but was able to induce shifts in community structure relative to the blank control. These shifts occurred in several taxa that could be considered favorable to the gut microbiome (e.g., increased *F. prausnitzii*). The shifts in community structure coincided with shifts in community function; thus, it is reasonable to conclude that an acute dose of AG1 could induce shifts in the gut microbiome. Subsequent clinical testing is needed to confirm whether these perceived favorable shifts translate to humans.

## Figures and Tables

**Table 1:** Results of the adonis on community structural heterogeneity.

Variable	Sum of Squares	R^2^	F	Adjusted *p*-Value
Treatment	0.2342	0.06964	14.4451	<0.001
Donor	2.6596	0.79093	82.0295	<0.001
Treatment/Donor	0.2743	0.08158	8.4608	<0.001
Residual	0.4689	0.13943		

**Table 2:** Results of the adonis on community functional heterogeneity using the GO Term database.

Variable	Sum of Squares	R^2^	F	Adjusted *p*-Value
Treatment	0.017889	0.07066	2.2808	0.034
Donor	0.110552	0.43667	7.0477	<0.001
Treatment/Donor	0.030612	0.12092	1.9515	0.025
Residual	0.094118	0.37176		

**Table 3:** Results of the adonis on community functional heterogeneity using the MetaCyc database.

Variable	Sum of Squares	R^2^	F	Adjusted *p*-Value
Treatment	0.12389	0.08555	1.7761	0.051
Donor	0.35297	0.24375	2.5301	<0.001
Treatment/Donor	0.13418	0.09266	0.9618	0.496
Residual	0.83704	0.57803		

## Data Availability

Data available upon reasonable request.
